# Factors associated with non-invasive mechanical ventilation failure in patients with hematological neoplasia and their association with outcomes

**DOI:** 10.1186/s40560-020-00484-x

**Published:** 2020-09-07

**Authors:** Lídia Miranda Barreto, Cecilia Gómez Ravetti, Thiago Bragança Athaíde, Renan Detoffol Bragança, Nathália Costa Pinho, Lucas Vieira Chagas, Fabrício de Lima Bastos, Vandack Nobre, on behalf of the Núcleo Interdisciplinar de Investigação em Medicina Intensiva (NIIMI)

**Affiliations:** 1 Hospital das Clínicas, Universidade Federal de Minas Gerais, Belo Horizonte, Brazil; 2 School of Medicine, Universidade Federal de Minas Gerais, Belo Horizonte, Brazil; 3 NIIMI (Interdisciplinary Nucleus of Investigation in Intensive Medicine), Federal University of Minas Gerais, Av. Professor Alfredo Balena, 190/533, Santa Efigênia, Belo Horizonte, Minas Gerais 30130-100 Brazil

**Keywords:** Hematological diseases, Mechanical ventilation, Non-invasive mechanical ventilation, Respiratory insufficiency, Intensive care unit (ICU)

## Abstract

**Background:**

The usefulness of non-invasive mechanical ventilation (NIMV) in oncohematological patients is still a matter of debate.

**Aim:**

To analyze the rate of noninvasive ventilation failure and the main characteristics associated with this endpoint in oncohematological patients with acute respiratory failure (ARF)*.*

**Methods:**

A ventilatory support protocol was developed and implemented before the onset of the study. According to the PaO_2_/FiO_2_ (P/F) ratio and clinical judgment, patients received supplementary oxygen therapy, NIMV, or invasive mechanical ventilation (IMV).

**Results:**

Eighty-two patients were included, average age between 52.1 ± 16 years old; 44 (53.6%) were male. The tested protocol was followed in 95.1% of cases. Six patients (7.3%) received IMV, 59 (89.7%) received NIMV, and 17 (20.7%) received oxygen therapy. ICU mortality rates were significantly higher in the IMV (83.3%) than in the NIMV (49.2%) and oxygen therapy (5.9%) groups (*P* < 0.001). Among the 59 patients who initially received NIMV, 30 (50.8%) had to eventually be intubated. Higher SOFA score at baseline (1.35 [95% CI = 1.12–2.10], *P* = 0.007), higher respiratory rate (RR) (1.10 [95% CI = 1.00–1.22], *P* = 0.048), and sepsis on admission (16.9 [95% CI = 1.93–149.26], *P* = 0.011) were independently associated with the need of orotracheal intubation among patients initially treated with NIMV. Moreover, NIMV failure was independently associated with ICU (*P* < 0.001) and hospital mortality (*P* = 0.049), and mortality between 6 months and 1 year (*P* < 0.001).

**Conclusion:**

The implementation of a NIMV protocol is feasible in patients with hematological neoplasia admitted to the ICU, even though its benefits still remain to be demonstrated. NIMV failure was associated with higher SOFA and RR and more frequent sepsis, and it was also related to poor prognosis.

## Introduction

Patients with hematological neoplasms hospitalized in intensive care units (ICU) present high mortality rates—50 to 70%—making the need of invasive mechanical ventilation (IMV) one of the main determining factors of this outcome. Acute respiratory failure (ARF) in these patients is multifactorial, and it is generally associated with disease progression, opportunistic infections, and treatment-associated toxicity [1–5]. Early use of non-invasive mechanical ventilation (NIMV) in patients with hematological neoplasms has been associated in some studies with a reduction in mortality and IMV requirement [1, 6–8]. Conversely, critically ill patients treated with NIMV that eventually get intubated apparently have a greater rate of complications, such as longer IMV duration, longer ICU stay, and higher mortality rate [8].

Given that, the aim of this study was to analyze factors associated with the transition from noninvasive to IMV (i.e., NIMV failure) in oncohematological patients with acute respiratory failure using a dedicated protocol.

## Methods

This was a prospective interventional study designed to test a ventilator assistance protocol in patients with hematological neoplasms admitted to an ICU with ARF. The hypothesis in this study was that the use of a protocol based on the best evidence in literature and on previous experience of the research team [9] would improve the suitability of NIMV recommendation in this group of patients. We also evaluated the characteristics associated with NIMV failure.

### Study population

This study was conducted in the adult ICU at the Hospital das Clínicas, a hospital that is managed by Universidade Federal de Minas Gerais (UFMG). This ICU is a medical and surgical unit, with 18 beds. Between January 2015 and January 2018, all the adult patients (age ≥ 18 years) with hematological neoplasms, admitted to the ICU with ARF, were evaluated regarding their eligibility to participate in this study and, when eligible, were included in one of the ventilatory assistance groups: oxygen therapy, NIMV, or IMV.

Inclusion criteria were (i) presence of acute respiratory failure (ARF) [1] initiated between the previous 48 h and admission to the ICU, associated with at least one of the following criteria: respiratory rate > 32/min, arterial oxygen saturation (SaO_2_) (< 94% in room air, P/F ratio < 300); (ii) signing of the informed consent form. Patients with a P/F ratio < 300 that had initially received NIMV were evaluated regarding of the associated factors and the consequences of NIMV failure. The present study was approved by the UFMG Research Ethics Committee, considering the terms set forth in Resolution 466/12 of the National Health Board, logged under protocol number CAAE 37297314.5.0000.5149. All included patients signed the informed consent form.

### Study procedures

The main demographic and clinical characteristics were collected upon inclusion, through a specific form created for this study: sex and age; hematological diagnosis; SAPS3 [10, 11]; APACHE II [12], and daily SOFA score [13] for the first 7 days of follow-up; presence of comorbidities; use of antifungals, antibiotics, and corticosteroid drugs; global leukocyte count; respiratory rate; and arterial blood gas analysis. During hospitalization, we collected data regarding patient follow-up, such as hemodialysis requirement, use of vasopressors, and chemotherapy in the ICU. In addition, all of the patients underwent daily laboratory exams, according to medical usual care, such as hemogram, serum urea, creatinine, arterial lactate and C-reactive protein (CRP) levels, arterial blood gas analysis, and measurement of NT-proBNP (only at inclusion). A radiography of the patient’s chest was taken of all the patients upon inclusion, while chest computed tomography (CT) and/or ultrasound were only taken according to medical team decision.

### Ventilatory assistance protocol

**Fig. 1:**
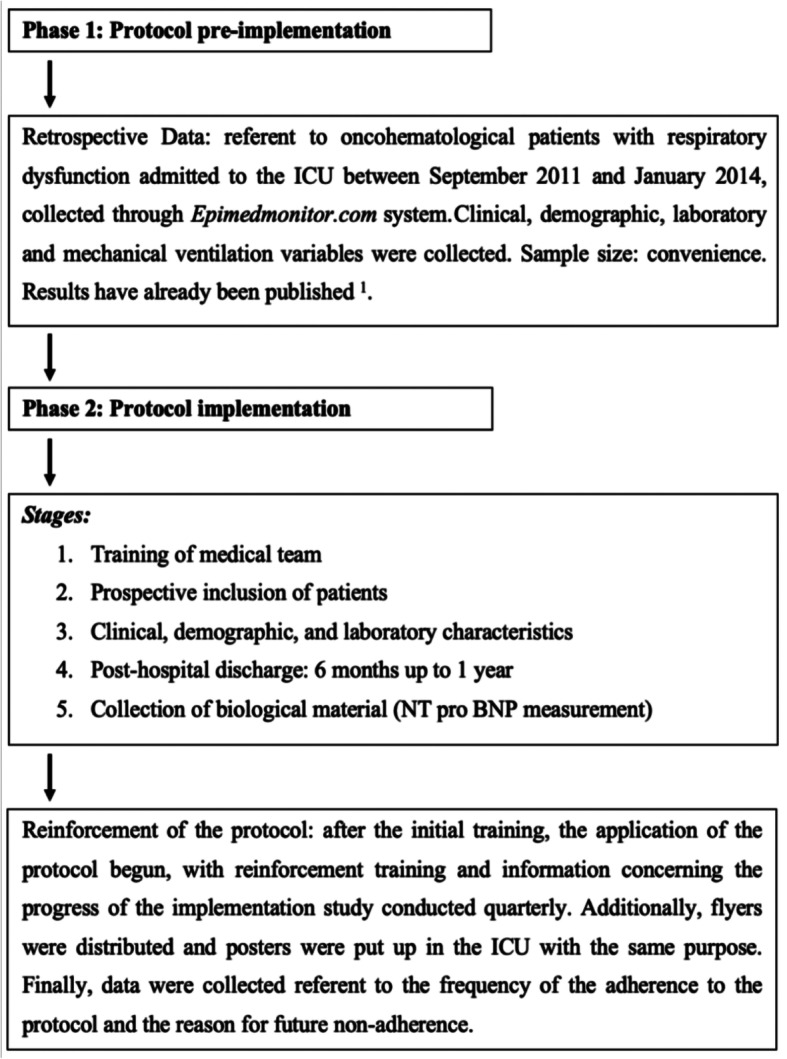
Implementation of the protocol for ventilatory assistance

The protocol of ventilatory assistance was developed specifically for this study (Fig. 1), based on information supplied by a retrospective [9] survey carried out at the same unit and on data from the literature [1, 14, 15]. In summary, the modality of ventilatory support was defined according to the P/F ratio observed at the moment of ICU admission. Thus, invasive mechanical support was strongly recommended (orotracheal intubation) if the P/F ratio was 100–150, while non-invasive support was recommended if the P/F ratio was between 200 and 300 in the absence of contraindications to this ventilator modality, as detailed below. If the P/F ratio was between 150 and 200, the protocol proposed IMV or NIMV according to the patient’s clinical condition, to gasometrical and radiological parameters, and to recommendations for each type of support. Supplementary oxygen therapy was recommended for patients with P/F ratio > 300, and they were monitored for any worsening on their clinical condition, namely, respiratory deterioration. Patients could be intubated or submitted to the NIMV according to the medical team’s decision. For patients submitted to NIMV, the characteristics associated with NIMV success or failures were evaluated as were the consequences of the eventual failures.

Even though the investigators made a recommendation according to the protocol, the final decision of ventilatory support for patients with P/F ratio between 150 and 200 was taken by the assistant medical team. Frequency with which these decisions coincided or not with the recommendations by the study protocol team were duly recorded.

### Implementation of the protocol

**Fig. 2:**
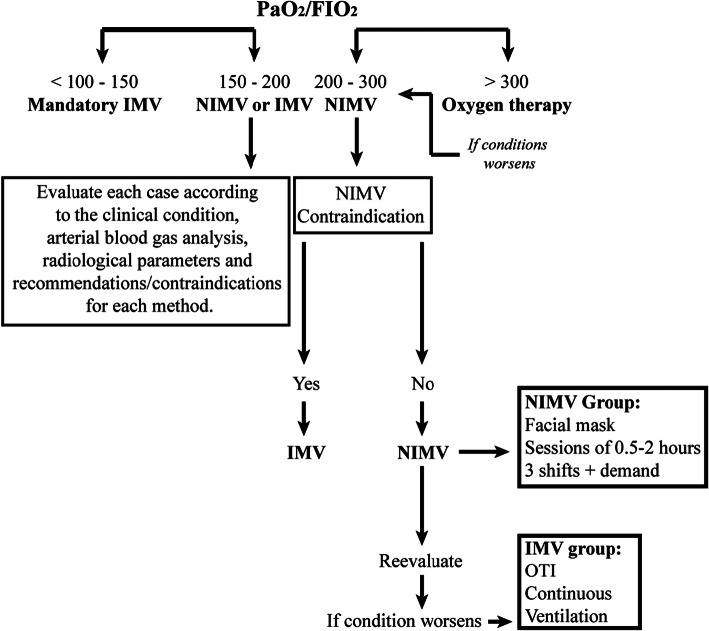
Flow chart of the ventilatory assistance protocol

The ventilatory assistance protocol was presented to ICU professionals (doctors, nurses, and respiratory therapists) who deal with mechanical ventilation, through an initial training session comprised of explanatory presentations, flyers fixed in the unit, and audiovisual resources. In addition, all cases were discussed daily with the medical staff, at the patient’s bedside. The presentations lasted approximately 15 min and were made by the main investigator. In parallel, meetings were held with the hematological team in an attempt to present and highlight the importance of the protocol, as well as seek ways to expedite the transfer to the ICU of potentially eligible patients. During the study period, ICU medical staff received monthly feedback on collected data and partial results, in addition to the sending, via email, of materials relevant to the study (Figs. 1 and 2).

### Noninvasive mechanical ventilation (NIMV)

NIMV was performed via facial or nasal mask—sessions of 0.5 to 2.0 h, at least three times a day (morning/afternoon/night) in the first 24 h after the patient’s ICU admission, and thereafter, as needed. This treatment was implemented by the respiratory therapy team, with the use of microprocessor mechanical ventilators, each with its own NIMV modality.

After explaining the procedure to the patient, he/she underwent a respiratory therapy session and was later placed in Fowler’s position (incline of 45°). Next, the interface was chosen, according to the best adaptation and comfort of the patient (nasal, facial, or total face) [16–19]. The following contraindications for NIMV were considered in the tested protocol: presence of hemodynamic instability with noradrenaline requirement in dose > 0.25 μg/kg/min, or in rapid ascension; Glasgow coma scale (GCS) < 14; recent upper digestive tract surgery (last 7 days) involving the esophagus, the stomach, and/or the duodenum; anastomosis or raphy; significant abdominal distension, with a high risk of aspiration; voluminous hemoptysis or epistaxis; unquestionable recommendation for IMV (ex., intense use of accessory muscles, signs of ventilatory muscle fatigue, shallow breathing, sweating).

The IMV was performed through an artificial airway (endotracheal tube). In these cases, mechanical ventilators in IMV screen mode were used, and the ventilatory mode was defined by the medical and respiratory therapy teams.

### Outcomes

Patients submitted to NIMV were considered for evaluation of the result, i.e., good response indicating progressive improvement of the respiratory dysfunction (success) or poor response, indicating the need of orotracheal intubation following a trial of NIMV (failure). Evaluated consequences to NIMV failure were ICU mortality, hospital mortality, and mortality occurring between 6 months and 1 year of follow-up. These outcomes were also measured in the entire population included in this study.

### Calculation of sample size

To define the impact of the tested protocol in the NIMV failure rate, the sample size was calculated based on the cohort of the prior study conducted by our research group [9]. In that study, we observed NIMV failure rate of 68.4%, resulting in orotracheal intubation. Considering a potentially more accurate recommendation for NIMV from the time point the protocol was implemented, we estimated that there would be an absolute reduction in approximately 30% of the NIMV failure rates, dropping from 68.4 to 40%. Considering the type I error (alpha) of 5% and the type II error (beta) of 20% (i.e., a power of 80%), allocation of 1:1, we reached a total sample of 96 patients to illustrate the benefits of this protocol.

### Statistical analysis

Patients’ adherence to the protocol is presented in percentage. Quantitative variables are expressed as average (± standard deviation—SD) or median (*P*25%*–P*75%), according to their distribution (normal or abnormal) measured by Shapiro-Wilk test. Comparative analyses were performed in regards of frequency, using the chi-squared test and Fisher exact test, as recommended, while the comparative analyses of the continuous variables were performed using Student’s *t* test or Mann-Whitney *U* test. A model of multivariate analysis was constructed to evaluate the association between NIMV failure and the outcome of death, adjusted for severity. Survival curves were constructed to analyze the time-dependent outcomes (mortality between 6 months and 1 year) by comparing the group of patients that failed and the group of patients that had success with NIMV, using the log-rank test.

The Cox proportional risk was used to perform the multivariate analysis comparing mortality and hospital stay between the NIMV failure and success groups. Variables with *P* < 0.2 in the bivariate analysis were included in this examination. Additionally, Poisson logistic regression model was performed, using the backward selection, to test the independent association of NIMV failure with ICU death and in outpatient follow-up. Kolmogorov-Smirnov test was used to test the calibration of the model. A value of *P* < 0.05 was considered to be statistically significant for all analyses, which were conducted in the SPSS program, version 23 (SPSS Inc., Chicago, IL, USA) and the Graphpad Prism program (version 2017).

## Results

One hundred and sixteen patients were evaluated and 34 (29.3%) were not included because they did not meet the eligibility criteria or died before 24 h of follow-up. Eighty-two patients were included in the final analysis.

The ventilatory assistance protocol was followed by the medical team in 78 (95.1%) patients. In four patients (4.9%) despite the protocol’s indication of NIMV as the first modality of ventilatory support, the ICU assistant team indicated IMV instead. The justification for the overruling of the protocol was recorded as “unfavorable clinical conditions for NIMV use despite the P/F ratios values, such as altered laboratory analysis, radiological worsening or rapid deterioration of clinical parameters.” No cases of NIMV intolerance were reported.

Considering the 82 patients included in the study, the average age was 52.1 ± 16 years and 44 (53.6%) of them were male. The hematological diagnoses were acute myeloid leukemia (*n* = 60, 73.2%), myelodysplasia (*n* = 8, 9.7%), lymphoma (*n* = 6, 7.3%), multiple myeloma (*n* = 5, 6.1%), and medullary aplasia (*n* = 3, 3.7%). Sixty-three (76.8%) patients had sepsis [20] at ICU admission, 76 (92.7%) were using antibiotics, and 60 (73.2%) were undergoing antifungal therapy.

During their ICU stay, 37 (45.1%) patients used chemotherapy, 31 (37.8%) received corticosteroids, and 8 (9.75%) underwent hemodialysis. The main comorbidities were heart failure (13.4%) and chronic obstructive pulmonary disease (9.7%).

Upon ICU admission, 6 (7.3%) patients were intubated and underwent IMV, 59 (72%) received NIMV, and 17 (20.7%) used supplementary oxygen therapy. ICU mortality was higher in the IMV group (83.3%) than in the NIMV (49.2%) and oxygen therapy (5.9%) groups (*P* < 0.001). Of note is that no difference was observed in the comparison among the three groups according to hospital mortality (*P* = 0.326) and 6 months to 1-year mortality (*P* = 0.284). In addition, the average NT-proBNP values were similar among the three subgroups (*P* = 0.711).

The characteristics of the 59 patients that initially received NIMV are presented in Table 1. From them, we gather that 30 (50.8%) had progressive worsening and were intubated after the NIMV trial (failure): 23 cases (76.6%) failed in the first 12 h, five (16.7%) between 12 and 24 h, and two (6.7%) after 24 h of ICU admission. Patients who failed to NIMV used vasopressors more often (90% vs. 27.5%, *P* < 0.001) and had higher values of SOFA at the time of ICU admission (7 [5-8.25] vs. 5 [4-7.], *P* = 0.007). In the multivariate analysis, sepsis upon ICU admission (16.9 [95% CI = 1.93–149.26], *P* = 0.011) and higher values of SOFA (1.35 [95% CI = 1.12–2.10], *P* = 0.007) and higher respiratory rate (1.10 [95% CI = 1.0–1.22], *P* = 0.048) were independently associated with an unsuccessful NIMV trial. Moreover, NIMV failure was independently associated with ICU mortality (OR = 1.62 [95% CI 1.29–2.03]; *P* < 0.001) and hospital mortality (OR = 1.29; [95% CI 1.01–1.64]; *P* = 0.049); the latter, even after adjustment for lactate, P/F ratio and respiratory rate upon ICU admission.

**Table 1:** Characteristics of patients submitted to NIMV as primary ventilatory assistance, stratified according to the response to this method (success or failure) in the prospective cohort

Characteristics (*n* = 59)	NIMV failure (*n* = 30)	NIMV success (*n* = 29)	*P*
Age (years)	50.2 (± 16.7)	53.4 (± 17.4)	0.48
Sex	17 (56.7%)	14 (48.3%)	0.51
Lactate	1.4 (1.1–3.7)	1.4 (1.1–2.2)	0.63
PaO_2_ upon inclusion (mmHg)	86.1 (73.7–115.9)	80.2 (74.2–109)	0.57
P/F upon inclusion	266.5 (222.8–336.3)	275 (243.5–335)	0.77
SaO_2_	90 (86.5–95)	92 (90–96.5)	0.11
Overall leukocyte count (cells/mm^3^)	1020 (150–4233)	1794 (650–9210)	0.07
CRP	261.6 (128.5–361.6)	169.3 (99.5–251.7)	0.09
RR (rpm)	34 (29–39)	29 (27.5–34)	0.042
APACHE II	21 (17–27)	21 (17.5–24)	0.53
SAPS 3	64.5 (53.7–71.2)	63 (55–71)	0.85
SOFA admission	7.0 (5.0–8.2)	5.0 (3.5–7.0)	0.007
Use of vasopressor	27 (90%)	8 (27.6%)	< 0.001
Use of ATB	28 (93.3%)	25 (86.2%)	0.36
Use of ATF	24 (80 %)	22 (75.9%)	0.70
Use of corticosteroids	10 (33.3%)	13 (44.9%)	0.36
NT-proBNP	3255 (1055–13727)	4650 (1440–11905)	0.516
Comorbidities			
COPD	4 (13.3%)	2 (6.9%)	0.42
HF	3 (10%)	7 (24.1%)	0.14
Follow-up data			
HD	3 (10%)	2 (6.9%)	0.66
CT	17 (56.7%)	12 (40%)	0.24
Exclusive palliative care	2 (6.7%)	6 (20.7%)	0.11

*ATB* antibiotics, *ATF* antifungal, *NT-proBNP* biomarker portion N-terminal of pro-hormone of B-type natriuretic peptide, *COPD* chronic obstructive pulmonary disease, *HF* heart failure, *PaO*_*2*_ arterial pressure of oxygen; *P/F* PaO_2_/FIO_2_ ratios, *ICU* intensive care unit, *NIMV* non-invasive mechanical ventilation, *SaO*_*2*_ oxygen saturation, *cells/mm*^*3*^ cells per cubic millimeter, *mmHg* millimeters of mercury, *CRP* reactive protein, *RR* respiratory rate (incursion per minute), *APACHE II* Acute Physiology and Chronic Health Evaluation II; *SAPS 3 Simplified Acute Physiology Score 3*, *SOFA Sequential Organ Failure Assessment*, *HD* hemodialysis, *CT* chemotherapy

Except for age, presented as mean ± standard deviation, all continuous variables are presented as median (Q1–Q3). Categorical variables are presented as *n* (%)

Significance was considered when *P* < 0.05

Similar results were observed in the analysis for survival measured after 6 to 12 months, which was higher among patients who experienced NIMV success, when compared to those who experience NIMV failure (OR = 1.32; [95% CI 1.07–1.64]; *P* < 0.001*)*, after adjustment for oxygenation index and respiratory rate.

**Fig. 3:**
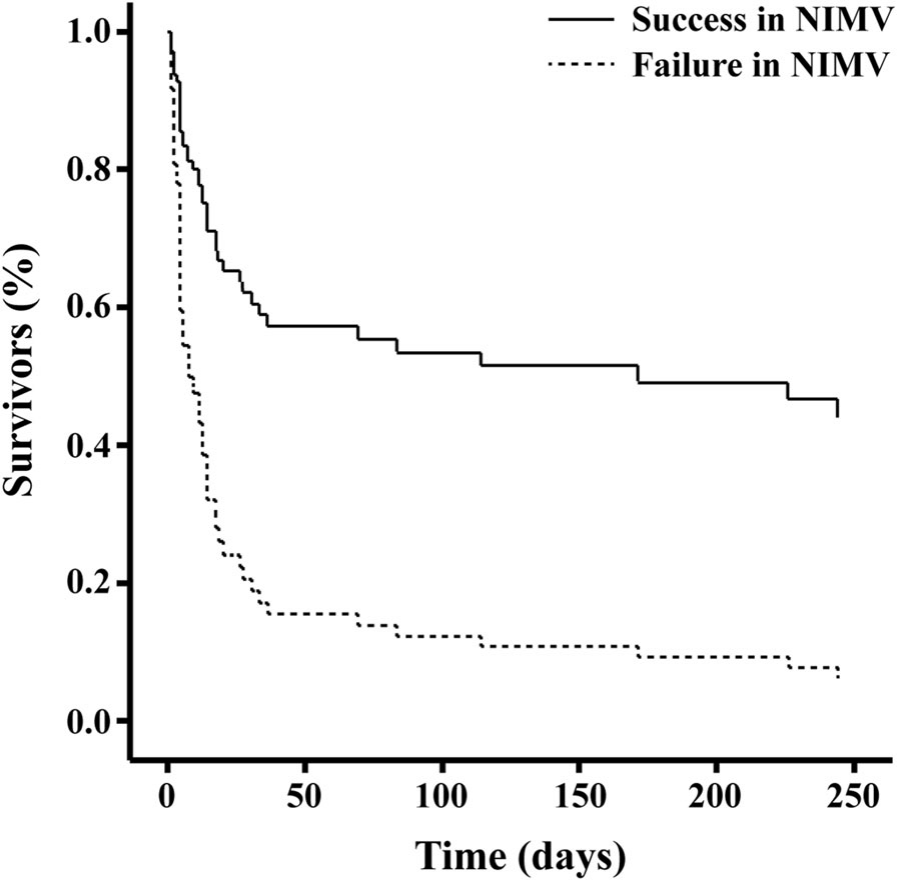
Cox model for NIMV failure, adjusted for the variable APACHE II, lactate, P/F ratios, and RR (*P* = 0.001, HR 3.0; 95% CI 1.58–5.8)

In the Cox model for the follow-up after 6 to 12 months, mortality was associated with NIMV failure (HR: 3.0; [95% CI 1.58–5.81]; *P* = 0.001), increased arterial lactate (HR: 1.2; [95% CI 1.10–1.52]; *P* = 0.002), and lower P/F ratio (HR: 1.0; [95% CI 1.00–1.01]; *P* = 0.03) (Fig. 3).

## Discussion

In this study, which evaluated the feasibility and benefits of a ventilator assistance protocol in adult oncohematological patients, we demonstrated a high adherence to such tested protocol. However, the protocol implementation was not associated with lower NIMV failure rates. Actually, the frequency of poor response and need of intubation after NIMV trial was similar to that found in other studies [6, 9, 14, 21–25], despite being lower than that observed in oncohematological patients admitted to the same unit (50.8% vs. 68.4%) in a previous study of our group [9]. In addition, patients with poor response to NIMV were apparently more severely ill, with higher median values of SOFA at baseline, and had a diagnosis of sepsis more frequently. Moreover, these patients had greater ICU and hospital mortality and a lower survival rate in the follow-up of 6 months to 1 year, as compared to NIMV success group. Overall, the ICU and hospital mortality rates observed in the present study were similar to those observed in other studies with oncohematological patients [21, 26].

Based on our results, we proposed some changes in the protocol currently used in our unit: If at least one of the severity factors is present (high SOFA score and RR or sepsis), IMV is strongly recommended.

In recent years, the survival of critically ill patients with hematological neoplasms has been improving most likely due to an early transfer of these patients to the ICU, and possibly to the use of non-invasive strategies, such as NIMV. However, the requirement of orotracheal intubation and IMV continues to be a determinant of poor prognosis in these patients [14, 27, 28]. In this sense, it is crucial to trace the criteria of recommendation for NIMV use and to attempt to understand which factors can predict an unsuccessful experience with this ventilatory support.

According to the current guideline from the American Thoracic Society (2017), the recommendation of implementing early NIMV for immunocompromised patients with ARF appears with a moderate degree of evidence [16]. Many studies sought out to investigate the factors associated with a poor or good response to NIMV in immunosuppressed patients [3, 4, 29], as well as the consequences of an eventual failure to this ventilatory modality. In agreement to our results, Belenguer-Muncharaz et al. retrospectively analyzed datasets from ICUs and observed high rates of complications, ICU and hospital mortality, and longer duration of IMV in patients in which NIMV failed, even when compared to patients who initially received IMV [1].

In the present study, vasopressors use was independently associated with NIMV failure. It is interesting to note that this association occurred even with the use of vasopressors in moderate doses, since the use of noradrenaline in doses of greater than 0.25 μg/kg/min was considered to be an exclusion criterion for the use of NIMV in this study. Finally, the use of vasopressors and the need for IMV were associated with higher rates of ICU mortality. In a historical cohort that was admitted in the same unit of this study, only the use of vasopressors upon inclusion was independently associated with NIMV failure [9, 14, 21].

In disagreement with our findings, Rathi et al. found no difference in mortality rates of patients submitted to early intubation, when compared to those who were initially submitted to NIMV and were subsequently intubated [30]. Likewise, Lemiale et al. compared the effects of NIMV and oxygen therapy on the mortality rates of patients with hematological neoplasms and found no difference in the mortality rates or in the intubation rates among these groups. The authors justified that the patients may have been less severe upon admission to the ICU [23]. In a multicenter study conducted in France, including oncohematological patients, Del Sorbo et al. showed no benefits in using NIMV in relation to mortality. Moreover, NIMV use was insufficient to delay or avoid orotracheal intubation in the studied population [31].

In the present study, no significant difference was observed in the serum levels of NT-pro-BNP among patients with poor or good response to NIMV. Similarly, no difference was found when the three modalities of initial ventilatory support were compared, i.e., IMV vs. NIMV vs. supplementary oxygen. Our hypothesis was that NT-proBNP could be able to identify patients with an excess of body and pulmonary fluids, who would supposedly present a better response to NIMV [32]. Previous studies have shown that patients with a clinical picture of pulmonary congestion can benefit from the association with NIMV and conventional clinical treatment, in turn maximizing the potential of its effects [33, 34]. The multiplicity of mechanisms involved in the ARF in the patients studied herein may partially explain our findings.

Another relevant point of this study was the evaluation of mortality over a longer period of time. Few studies have tested this outcome in critically ill oncohematological patients. Richards et al. observed that the need for IMV, NIMV failure, severity of the hematological disease, and recent bone marrow transplant were independently associated with higher rates of mortality in 6 months [35].

This study has some limitations that should be considered. It was a single-center study and the predicted sample size was not reached, which limits the internal validation and the generalizability of our findings for other settings. In addition, we were not able to include a control group of non-protocol-guided ventilatory assistance. Besides logistical constraints, we considered doing so could be unethical since the tested protocol was based on the best evidence available in the literature and on our own experience in this field. Finally, the tested protocol did not include high-flow nasal cannula oxygen therapy, which has been tested with promising, yet controversial results in some studies.

## Conclusion

Higher SOFA score at baseline, as well as higher respiratory rate and sepsis at the time of ICU admission, was independently associated with the need of orotracheal intubation among patients initially treated with NIMV. Moreover, patients in whom NIMV failed had a worse prognosis than did those who had a successful experience with this ventilatory modality. The identification of the ideal NIMV candidates among oncohematological patients can be useful to better take advantage of this tool in cases of respiratory dysfunction occurring in this population.

## Data Availability

The datasets used and/or analyzed during the current study are available from the corresponding author on reasonable request.

## Abbreviations

ATB: Antibiotics
ATF: Antifungal
NT-proBNP: Biomarker portion N-terminal of pro-hormone of B-type natriuretic peptide
PaO_2_: Arterial pressure of oxygen
ICU: Intensive care unit
SaO_2_: Oxygen saturation
cells/mm^3^: Cells per cubic millimeter
CRP: C-reactive protein
RR: Respiratory rate (incursion per minute)
APACHE II: Acute Physiology and Chronic Health Evaluation II
*N*: Sample
SOFA: *Sequential Organ Failure Assessment*
SAPS 3: *Simplified Acute Physiology Score 3*
P/F: PaO_2_/FIO_2_ ratios
COPD: Chronic obstructive pulmonary disease
HF: Heart failure
CT: Chemotherapy
HD: Hemodialysis
TCT: Tracheostomy
CPA: Cardiopulmonary arrest
ARDS: Acute respiratory distress syndrome
O_2_: Supplementary oxygen therapy
NIMV: Non-invasive mechanical ventilation
IMV: Invasive mechanical ventilation
